# Role of Pseudouridine Formation by Deg1 for Functionality of Two Glutamine Isoacceptor tRNAs

**DOI:** 10.3390/biom7010008

**Published:** 2017-01-26

**Authors:** Roland Klassen, Raffael Schaffrath

**Affiliations:** Institut für Biologie, Fachgebiet Mikrobiologie, Universität Kassel, Heinrich-Plett-Str. 40, D-34132 Kassel, Germany

**Keywords:** tRNA modification, *PUS3*/*DEG1*, translation, 5-methoxycarbonylmethyl-2-thiouridine, non-sense suppression, *sup70-65*

## Abstract

Loss of Deg1/Pus3 and concomitant elimination of pseudouridine in tRNA at positions 38 and 39 (ψ38/39) was shown to specifically impair the function of tRNA^Gln^_UUG_ under conditions of temperature-induced down-regulation of wobble uridine thiolation in budding yeast and is linked to intellectual disability in humans. To further characterize the differential importance of the frequent ψ38/39 modification for tRNAs in yeast, we analyzed the in vivo function of non-sense suppressor tRNAs *SUP4* and *sup70-65* in the absence of the modifier. In the tRNA^Tyr^_GψA_ variant *SUP4*, UAA read-through is enabled due to an anticodon mutation (UψA), whereas *sup70-65* is a mutant form of tRNA^Gln^_CUG_ (*SUP70*) that mediates UAG decoding due to a mutation of the anticodon-loop closing base pair (G31:C39 to A31:C39). While *SUP4* function is unaltered in *deg1/pus3* mutants, the ability of *sup70-65* to mediate non-sense suppression and to complement a genomic deletion of the essential *SUP70* gene is severely compromised. These results and the differential suppression of growth defects in *deg1* mutants by multi-copy *SUP70* or *tQ(UUG)* are consistent with the interpretation that ψ38 is most important for tRNA^Gln^_UUG_ function under heat stress but becomes crucial for tRNA^Gln^_CUG_ as well when the anticodon loop is destabilized by the *sup70-65* mutation. Thus, ψ38/39 may protect the anticodon loop configuration from disturbances by loss of other modifications or base changes.

## 1. Introduction

In addition to standard nucleosides, tRNA is known to contain a large variety of modified nucleosides, formed by the addition of chemical groups or isomerization. Pseudouridine (ψ) is an isomer of uridine (5-ribosyl-uracil) and represents the initially discovered and most abundant modified nucleoside present in all three domains of life [1,2]. The model organism *Saccharomyces cerevisiae* has ten characterized pseudouridine synthases (Pus1–9 and Cbf5) that modify cytoplasmic and mitochondrial tRNAs and/or other types of RNA, including rRNA, small nuclear RNA (snRNA) and mRNA [2]. Despite its ubiquitous presence in tRNA, ψ appears to affect tRNA function rather subtly, since it is not generally required for cell viability, as demonstrated by the non-essential nature of all of the *PUS* genes in the yeast system [3]. However, the absence of *PUS3/DEG1* in yeast uniquely causes slow growth, in particular at elevated temperature [4]. The general effects of ψ on tRNA function are likely mediated by increasing the rigidity of the sugar phosphate backbone and base stacking [2,5]. In humans, homozygous mutation of the *DEG1* orthologue *PUS3* is correlated with intellectual disability, suggesting a significant contribution to tRNA functioning not only in yeast but also in humans [6].

In yeast, Deg1 dependent formation of ψ occurs in a majority of tRNAs either at position 38 or 39 [4,7]. Slow growth of yeast mutants lacking Deg1 is strongly aggravated if parts of the wobble uridine modification 5-methoxy-carbonylmethyl-2-thiouridine (mcm^5^s^2^U) are simultaneously absent [8,9]. The mcm^5^ component of the mcm^5^s^2^U modification is formed by a pathway requiring the Elongator complex and a variety of accessory and potentially regulatory proteins [10,11,12,13,14,15,16,17,18,19]. The separate Urm1 pathway mediates the transfer of sulfur to the carbon 2 of the uracil base. This requires the sulfur carrier and ubiquitin-like modifier protein Urm1 and a number of additional proteins operating upstream (Nfs1, Tum1, Uba4) and downstream (Ncs2, Ncs6) of Urm1 resulting in sulfur flow from cysteine to mcm^5^U [11,20,21,22,23,24,25]. The absence of either part alone (mcm^5^U or s^2^U) induces shared pleiotropic phenotypes, including growth defects at elevated temperature and sensitivity to a number of exogenous stressors including the target of rapamycin complex 1 (TORC1)-inhibiting drug rapamycin [26,27]. On a functional level, partial loss of mcm^5^s^2^U reduces binding of tRNA to the ribosomal A-site and in turn causes reading frame slippage [28,29]. Combined loss of mcm^5^ and s^2^U increases the severity of these phenotypes and causes a defect in protein homeostasis resulting in the accumulation of protein aggregates [30,31,32]. Similarly, combination of Elongator or Urm1 pathway mutations with a *deg1* deletion aggravates growth defects and induces protein aggregation, which is further linked to severe problems in cytoskeleton organization, cell polarity and nuclear segregation [9]. 

Pleiotropic phenotypes of mutants lacking ψ38/39 together with mcm^5^s^2^U can be specifically rescued by overexpressing tRNA^Gln^_UUG_, which is reasonable since this tRNA alone carries both modifications [8,9]. In addition to tRNA^Gln^_UUG_, there is an isoacceptor tRNA with the anticodon sequence CUG that is thought to decode the G-ending codon for Gln. Genetic data from yeast suggests that the U34-containing isoacceptor mainly decodes the A-ending codon and only inefficiently reads the G-ending codon [33]. Because of this, the single *tQ(CUG)* gene has been found to be essential for yeast cell viability [33]. Except for the wobble nucleoside and a variable base (A/G) in position 42, tRNA^Gln^_CUG_ contains an anticodon stem loop (ASL) identical with that of tRNA^Gln^_UUG_ and is also modified by Deg1 at position U38 [34]. Interestingly, however, growth defects of *deg1* single mutants were found to be suppressible by overexpression of tRNA^Gln^_UUG_, but not tRNA^Gln^_CUG_. This raises the possibility of differential functional dependencies of the two Gln isoacceptor tRNAs on the presence of ψ38. In this study, we investigated potential reasons for such divergent effects of one and the same modification on two highly similar tRNA species.

## 2. Results and Discussion

### 2.1. Phenotypic Rescue of *deg1* Mutants by tRNA Overexpression

**Figure 1:**
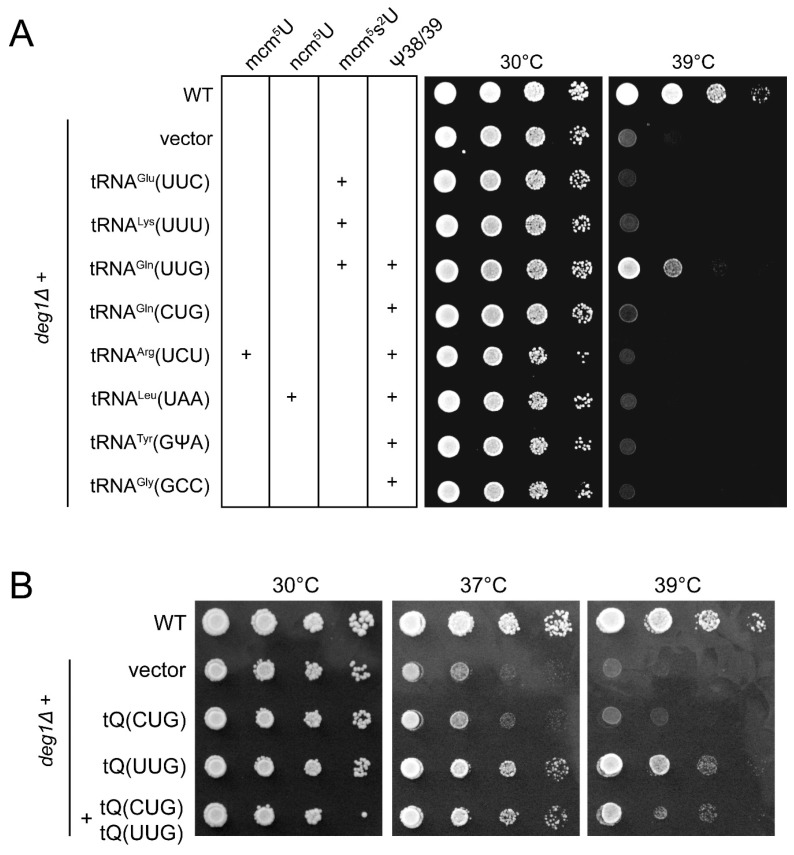
Rescue of *deg1* growth defect by tRNA overexpression. (**A**) Single tRNA overexpression. Indicated tRNA species were expressed from multicopy plasmids and in *deg1* mutants and serial dilutions spotted on yeast peptone dextrose (YPD) plates that were incubated at 30 °C or 39 °C. In wild type cells, some tRNA species selected for overexpression carry 5-methoxy-carbonylmethyl-2-thiouridine (mcm^5^s^2^U), 5-carbamoylmethyluridine (ncm^5^U), 5-methoxy-carbonylmethlyuridine (mcm^5^U), pseudouridine (ψ) 38/39 or combinations thereof, as indicated by (+); (**B**) Combined overexpression of tRNA^Gln^_UUG_ and tRNA^Gln^_CUG_. Cells carrying either a multicopy construct for overexpression of tRNA^Gln^_UUG_ (tQ(UUG)), tRNA^Gln^_CUG_ (tQ(CUG)) or both were assayed as in (**A**) for growth at 30, 37 and 39 °C. WT: wild type.

tRNA^Gln^_UUG_ functionality has been shown to depend on ψ38, in particular under conditions of heat stress [8]. Since the tRNA^Gln^_CUG_ isoacceptor harbours an ASL almost identical to that of tRNA^Gln^_UUG_, we first investigated whether there are indeed differential dependencies of the two Gln isoacceptors on the presence of ψ38. The thermosensitive growth phenotype of *deg1* mutants was shown to be specifically suppressible by overexpression of tRNA^Gln^_UUG_ but not tRNA^Gln^_CUG_ or any other tRNA carrying U38 or U39, which is modified by Deg1 to ψ [8]. We confirm this result and find a clear suppression of *deg1*-induced thermosensitivity by multicopy tRNA^Gln^_UUG_ but not tRNA^Gln^_CUG_ or other tester tRNAs (Figure 1A). However, rescue by multicopy tRNA^Gln^_UUG_ is clearly incomplete as growth at 39 °C is not fully restored to wild type levels (Figure 1A). Therefore, we checked whether co-overexpression of the isoacceptor tRNA^Gln^_CUG_ together with tRNA^Gln^_UUG_ improves growth at elevated temperature (39 °C) compared to overexpression of tRNA^Gln^_UUG_ alone. We find that under these conditions, growth of *deg1* mutants already overexpressing tRNA^Gln^_UUG_ is not further improved by additional overexpression of tRNA^Gln^_CUG_ (Figure 1B). These results suggest that under heat stress conditions, growth defect of yeast cells lacking ψ38 results almost exclusively from malfunction of tRNA^Gln^_UUG_ rather than tRNA^Gln^_CUG_, despite containing almost identical ASLs.

It should also be taken into account that copy numbers of the two Gln isoacceptors in yeast differ significantly due to the number of the respective tRNA genes (nine copies of *tQ(UUG)* versus one copy of *tQ(CUG)* (*Saccharomyces cerevisiae* genome database)). This correlates with a biased codon usage and may impact on the efficiency of phenotypic suppression by tRNA overexpression. Based on this consideration, phenotypic effects of the *DEG1* deletion may be rescuable by overexpression of tRNA^Gln^_UUG_, since this is the major Gln isoacceptor and consequently more important for overall translation as compared to the minor tRNA^Gln^_CUG_. However, the single *tQ(CUG)* gene is in fact known to be essential for yeast viability [33], indicating that this C34-containing tRNA is indeed indispensable for translation, despite its lower frequency of utilization compared to the one containing mcm^5^s^2^U34. Hence, the differential dependency of both isoacceptors on ψ38 as indicated by differential phenotypic suppression of the *deg1* phenotype is presumably, at least in part, due to the difference at the wobble base. Interestingly, tRNA^Gln^_UUG_ is known to harbour a hypomodified wobble uridine under mild heat stress conditions, which results from a destabilization of the *URM1* pathway and in turn suppresses the tRNA thiolation reaction that converts mcm^5^U to mcm^5^s^2^U [8,25,35,36,37]. Thus, an explanation why tRNA^Gln^_UUG_ depends more than tRNA^Gln^_CUG_ on ψ38 under heat stress conditions might be the presence of an additional functional impairment of the former tRNA due to wobble uridine hypomodification. tRNA^Gln^_CUG_ carries a different unmodified wobble base (C34) and therefore remains unaffected by the temperature effect on wobble uridine thiolation.

### 2.2. Genetic Interactions of *DEG1* under TORC1 Inhibition

**Figure 2:**
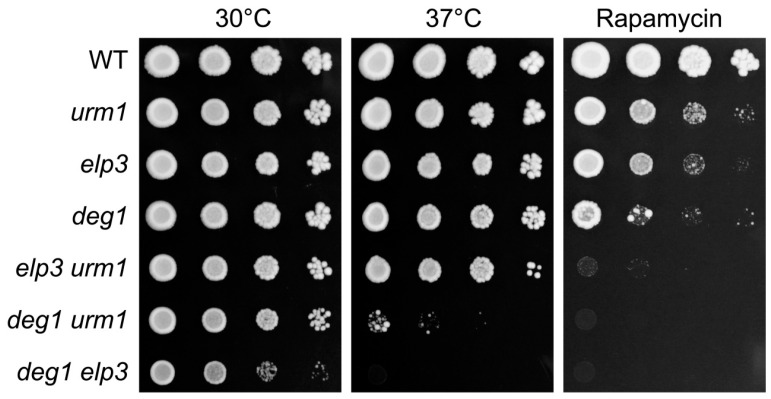
Interactions between *DEG1* and genes coding for Elongator (*ELP3*) or Urm1 (*URM1*) pathway components under TORC1 inhibition. Indicated single and double mutants were spotted on drug-free yeast peptone dextrose (YPD) plates which were incubated at 30 °C or 37 °C or YPD medium containing 5 nM of the TORC1 inhibiting drug rapamycin.

In support of a specific requirement for ψ38 in tRNA^Gln^_UUG_ in the event of wobble base hypomodification, strong synthetic growth defects were observed upon combining the *deg1* mutation with mutations in Elongator or Urm1 pathway genes, indicative of functional crosstalk between the modifications at position 34 and 38 in tRNA^Gln^_UUG_ [8,9,38]. Elongator and Urm1 pathway mutants are further known to exhibit shared phenotypes, including translational inaccuracy and sensitivity to TORC1 inhibiting agents such as caffeine or rapamycin and these are synergistically increased in double mutants lacking both modification activities [9,30,31,32]. To check whether negative genetic interactions between *DEG1*, Elongator and *URM1* can be extended to conditions of TORC1 inhibition, we scored rapamycin sensitivity of *deg1*, *urm1* and *elp3* single and all possible double mutant combinations. As shown in Figure 2, there is a clear rapamycin sensitivity in all three single mutants and this sensitivity is strongly aggravated in all the double mutants, with stronger effects seen in *elp3 deg1* and *urm1 deg1*, where U34 and U38/39 are hypomodified. Thus, there is an overlapping phenotype in *deg1*, *elp3* and *urm1* mutants and all affected modifications (mcm^5^/s^2^U, ψ38/39) appear to mediate rapamycin tolerance independently of each other, possibly by maintaining a related aspect of tRNA function. In particular, the observed rapamycin sensitivity of *deg1* single mutants in the absence of heat stress suggests that ψ38 alone becomes important for tRNA function under at least one stress condition that is different from heat. Since there is strong negative genetic interaction on rapamycin between *DEG1* and *URM1*/Elongator, the latter of which affect tRNA^Gln^_UUG_ but not tRNA^Gln^_CUG_, we assume that these effects are caused by specific malfunction of tRNA^Gln^_UUG_.

### 2.3. Replacement of *tQ(CUG)* by the *sup70-65* Allele in Haploid Yeast

**Figure 3:**
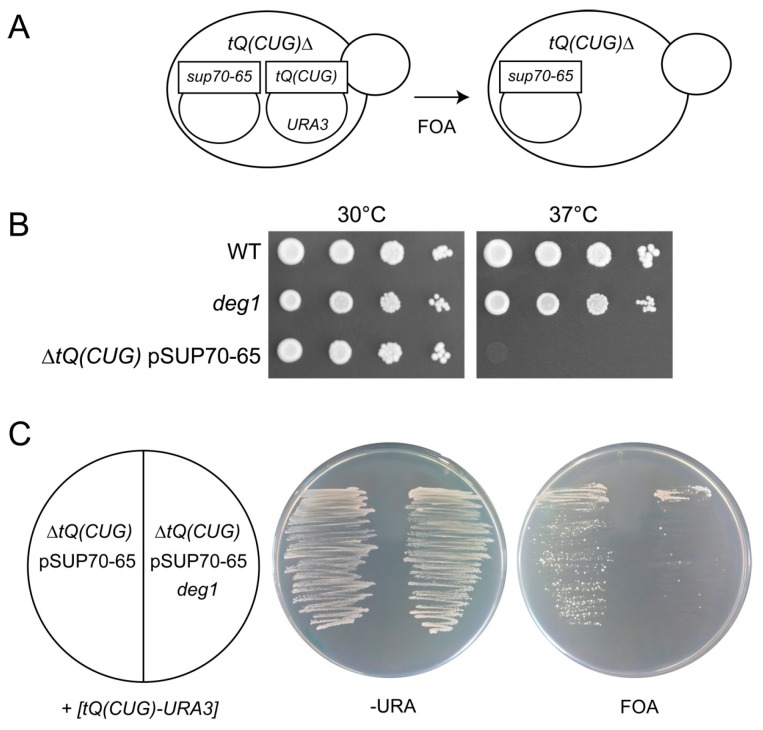
Role of ψ 38 for the *sup70-65* allele of the *tQ(CUG)* gene. (**A**) Experimental design for the construction of a haploid *sup70-65* strain and plasmid shuffle. A strain carrying a genomic deletion of the essential single-copy *tQ(CUG)* is kept alive by plasmid pAK1 (*tQ(CUG)-URA3*). A second plasmid is introduced carrying the mutant *sup70-65* allele of *tQ(CUG)* and pAK01 is eliminated from the strain by growth on 5-fluoro-orotate (5-FOA) media; (**B**) Drop-dilution assay as in Figure 1 with WT , *deg1* and the haploid strain carrying *sup70-65* as the sole genetic source of *tQ(CUG)*; (**C**) Plasmid shuffle assay to check for the ability to lose pAK01 (*tQ(CUG)-URA3*) in the presence (right) or absence (left) of an additional *deg1* deletion.

The finding that tRNA^Gln^_UUG_ lacking ψ38 becomes vulnerable to disturbances of the natural anticodon loop configuration (loss of U34 modification) suggested that tRNA^Gln^_CUG_ devoid of ψ38 might also be sensitized to destabilization of the ASL. However, as C34 is unmodified and no modification except ψ38 is known for the ASL of tRNA^Gln^_CUG_ [7,34], we utilized a variant of tRNA^Gln^_CUG_ with a disturbance of the ASL due to a sequence change (G31A). This mutation, known as the *tQ(CUG)* allele *sup70-65*, replaces the Watson–Crick base pair G31:C39 normally closing the anticodon loop of tRNA^Gln^_CUG_ by a mismatch (A31:C39) and reduces stability and charging efficiency of the mutant tRNA [39,40,41]. Despite these strong effects on the tRNA product of the essential single-copy gene *tQ(CUG)*, diploid yeast cells carrying the homozygous *sup70-65* mutation are viable [39,40,41] and may provide an opportunity to analyze the effects of ψ38 removal on tRNA^Gln^_CUG_ in the presence of a destabilized ASL. We utilized a haploid strain (S288C) lacking the single genomic copy of *tQ(CUG)* and carrying the wild type *tQ(CUG)* gene on a plasmid that is counter-selectable by 5-fluoro-orotate (5-FOA) [31,40]. This strain was transformed with a second centromeric plasmid carrying the *sup70-65* allele of the *tQ(CUG)* gene. Subsequently, the former plasmid was removed by growing cells on 5-FOA medium, resulting in a strain carrying *sup70-65* as the sole genetic source of *tQ(CUG)* (Figure 3A). The viability of this strain suggests that even in haploid S288C cells, the mutant form of *tQ(CUG)* is active to maintain sufficient levels of translation. However, we find that in comparison to wild type, a strong thermosensitivity is caused by the mutation, while no significant growth defect was observed at 30 °C (Figure 3B). This finding possibly indicates that the destabilized form of tRNA^Gln^_CUG_ (*sup70-65*) is sufficiently active in translation at normal, but not at elevated temperatures. The absence of defects in growth or CAG decoding in diploid *sup70-65* cells was already documented [39]. However, more recent work demonstrated a reduced expression of reporter genes with tandem CAG codons and several natural CAG-codon-containing mRNAs [40,41]. Hence, a translational defect is already present in *sup70-65* cells at 30 °C and this may be aggravated at elevated temperatures, as evidenced by the observed growth defect (Figure 3B). It is noteworthy that tRNA^Gln^_CUG_ can be functionally replaced by the inefficient CAG decoder tRNA^Gln^_UUG_ if the latter is present at elevated levels and carries the full mcm^5^s^2^U modification [33]. In such strain, CAG codons are exclusively decoded by tRNA^Gln^_UUG_, involving U-G wobble and the mcm^5^s^2^U modified wobble base. In particular, the absence of s^2^U in tRNA^Gln^_UUG_ entirely prevents the suppression of lethal effects of a *tQ(CUG)* deletion by multi-copy *tQ(UUG)* [33]. Thus, the strong growth defect of haploid *sup70-65* cells at elevated temperatures may also indicate that tRNA^Gln^_UUG_ contributes, to some extent, to CAG (Gln) decoding in this mutant, since tRNA^Gln^_CUG_ is functionally impaired. If so, elevated temperature would repress thiolation and in turn prevent the engagement of tRNA^Gln^_UUG_ in decoding of the G-ending Gln codon (CAG).

To test whether *sup70-65* is still able to replace wild type *tQ(CUG)* when ψ38 is removed, we deleted the *DEG1* gene in a strain carrying a genomic deletion of *tQ(CUG)* and containing the wild type *tQ(CUG)* gene on the counter-selectable plasmid. Then, the ability to lose the *tQ(CUG)* plasmid in the presence of the *sup70-65* plasmid was assayed in comparison to the strain lacking the *deg1* mutation. We find that colony formation on 5-FOA medium, which counter-selects the *tQ(CUG)* wild type allele, is severely reduced in the strain lacking *DEG1* (Figure 3C). This result supports the interpretation that ψ38 becomes critical for function of tRNA^Gln^_CUG_ in the presence of a destabilized ASL.

### 2.4. Comparison of Non-Sense Suppression by *sup70-65* and *SUP4* in the Presence and Absence of ψ38/39

**Figure 4:**
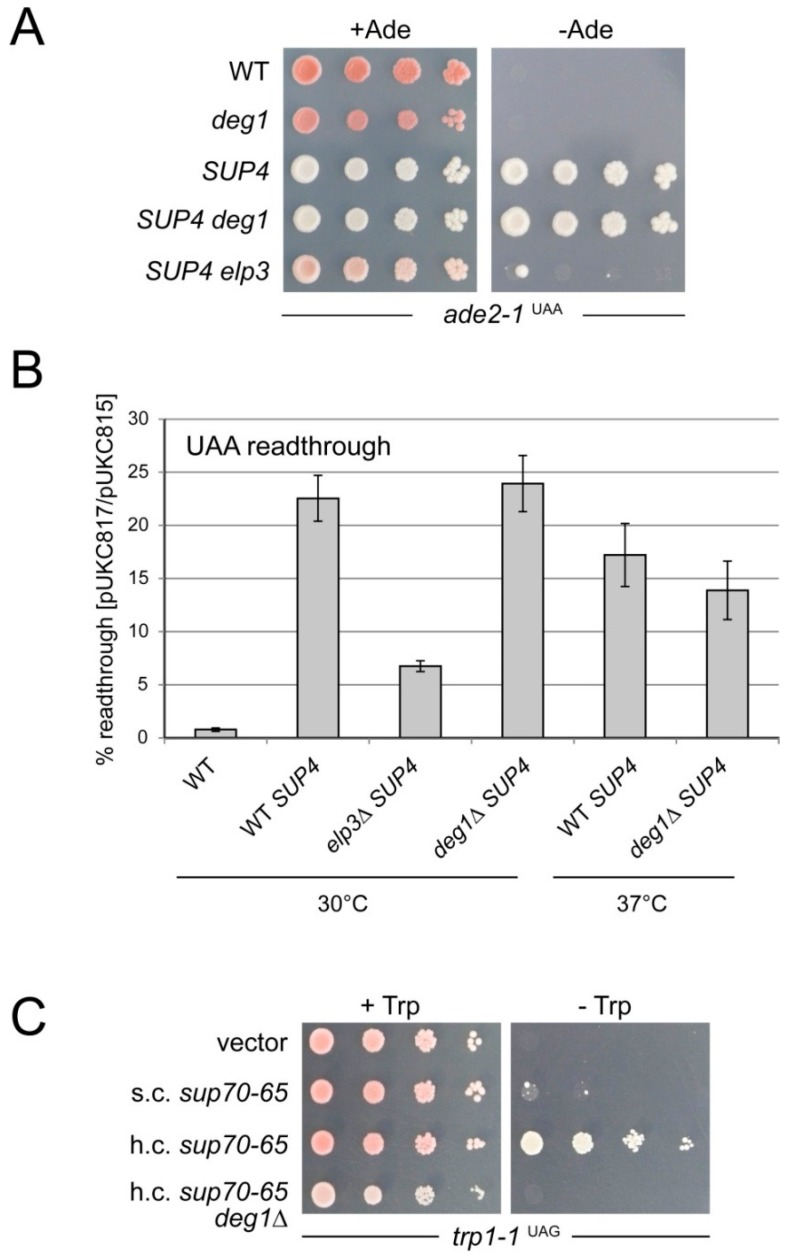
Role of ψ 38/39 for non-sense suppression by *SUP4* or *sup70-65*. (**A**) *SUP4*-mediated read-through of *ade2-1*. WT: Strain W303-1B, lacking *SUP4*; *deg1*: *DEG1* deletion in W303-1B; *SUP4*: strain UMY2893, carrying *SUP4*; *SUP4 deg1*: deletion of *DEG1* in UMY2893; *SUP4 elp3*: deletion of *ELP3* in UMY2893. Serial dilutions were spotted on adenine-free (-Ade) or adenine-containing medium (+Ade); (**B**) Quantitative *SUP4*-mediated *lacZ* read-through assay. Strains as described in (**A**) were transformed with pUKC815 and pUKC817 and relative read-through levels measured as described in Materials and Methods. 30 °C and 37 °C indicate growth temperatures. Averages of at least three biological replicates are shown along with standard deviation; (**C**) *sup70-65*-mediated read-through of *trp1-1*. Strain W303-1B or W303-1B *deg1* was transformed with empty vector (vector), single-copy (s.c.) or high-copy (h.c.) constructs with the *sup70-65* allele of *tQ(CUG)*. The ability of strains to grow on tryptophan-free (-Trp) medium by read-through of the *trp1-1* marker was scored by drop dilution assays. +Trp: tryptophan-containing medium condition.

Since *sup70-65* is known as an amber suppressor [39,40], we analyzed how loss of ψ38 influences the efficiency of UAG stop codon read-through by *sup70-65*. If ψ38 is indeed crucial for tRNA^Gln^_CUG_ function in the presence of a destabilized ASL, then stop codon read-through by *sup70-65* could be impaired by the *deg1* mutation. As a comparison, we scored the efficiency of *SUP4*, a distinct non-sense suppressor tRNA, derived from tRNA^Tyr^_GψA_ [10]. *SUP4* can read-through ochre (UAA) stop codons due to a base change at the wobble position and was already shown to crucially depend on the presence of the Elongator-mediated mcm^5^U modification (mcm^5^UψA) [10]. Since tRNA^Tyr^_GψA_ harbours a *DEG1* dependent ψ in position 39 [7], *SUP4* could in principle also be affected by the *deg1* mutation. To test this, we utilized a W303-1B-derived strain carrying *SUP4* and *ade2-1*, an allele of *ADE2* causing adenine auxotrophy due to an ochre mutation [10]. Non-sense suppression of the *ade2-1* transcript is known to cause adenine prototrophy and change of colony color from red (no read-through) to white (read-through) [10]. In this background, we deleted the *DEG1* gene and scored adenine auxotrophy as well as color colony in direct comparison with an *elp3* mutant that is known to affect *SUP4*-mediated read-through. While the presence of *SUP4* causes the expected change in color and adenine prototrophy, we find that this indeed depends on a functional *ELP3* gene but not on *DEG1* (Figure 4A). Since this assay might be too insensitive to detect smaller reductions in *SUP4*-mediated read-through and to confirm the obtained result with a premature ochre codon in another reporter gene, we utilized a quantitative assay relying on a *lacZ* reporter with an engineered ochre codon [33,42]. Again, read-through in this reporter was strongly enhanced by the presence of *SUP4* and suppressed by the *elp3* mutation, yet not by *deg1* (Figure 4B). Further, even application of mild temperature stress (37 °C) to cells carrying the reporter constructs did not significantly reduce read-through levels of the *deg1* mutant as compared to the wild type *SUP4* strain under the same conditions (Figure 4B). Thus, Deg1-mediated ψ39 in itself does not detectably impact on the function of *SUP4* in non-sense suppression in two different read-through contexts. This result is consistent with the absence of suppression of temperature sensitive growth of the *deg1* mutant by multi-copy tRNA^Tyr^_GψA_ (Figure 1A) and supports the conclusion that ψs formed by Deg1 in distinct tRNA species may have different functional consequences. 

For *sup70-65*-mediated non-sense suppression, we utilized strain W303-1B, which carries the *trp1-1* allele that contains a premature amber (UAG) codon and is efficiently suppressible by *sup70-65* when the mutant tRNA gene is present on a multi-copy plasmid [40]. We deleted the *DEG1* gene in W303-1B and introduced *sup70-65* on a multi-copy construct. As a control, we used the parental *DEG1* wild type strain and introduced *sup70-65* on both, single and multi-copy vectors [40]. While there is no major difference in growth on tryptophan-supplemented minimal media, the presence of the *sup70-65* gene in both single and multi-copy conferred an ability to grow on tryptophan-free media specifically to the *DEG1* wild type. As expected, multi-copy expression of *sup70-65* was required for induction of efficient growth in the absence of tryptophan, due to relatively inefficient amber suppression by *sup70-65* via first base wobble (U1:G36/A2:U35/G3:C34) [40]. In the absence of the suppressor tRNA, no growth on tryptophan-free medium was observed and, most importantly, growth was also absent in the *deg1* mutant with the multi-copy *sup70-65* construct causing efficient read-through in the *DEG1* wild type strain. Thus, indeed *sup70-65*-mediated non-sense suppression critically depends on the presence of ψ38 formation by Deg1.

## 3. Materials and Methods

### 3.1. Strains, Plasmids and General Methods

Yeast strains used are listed in Table 1. Standard methods were used in this study for yeast growth [43]. Yeast was routinely grown in complex yeast peptone dextrose (YPD) medium. To select transformants or maintain plasmids, either synthetic complete (SC) medium lacking specific nutrients or YPD medium containing 200 µg·mL^−1^ G418 was used. Deletion of *DEG1* involved PCR-mediated synthesis of deletion cassettes using previously described oligonucleotides [9] and template plasmids pUG6 and pUG27 [44]. Transformation was done using the PEG/lithium acetate method [45]. Correct genomic replacement of the *DEG1* gene was verified by PCR using oligonucleotides described [9]. Multicopy plasmids for overexpression of tRNA^Glu^_UUC_, tRNA^Lys^_UUU_, tRNA^Arg^_UCU_, tRNA^Leu^_UAA_, tRNA^Tyr^_GUA_ and tRNA^Gly^_GCC_ were described earlier [13] as well as the construct for tRNA^Gln^_UUG_ [46]. Wild type tRNA^Gln^_CUG_ overexpression used 2 µ plasmid pSUP70 [40]. pAK01 is a single copy (CEN/ARS) plasmid carrying the wild type *tQ(CUG)* gene as well as *URA3* as a counter-selectable marker [40]. Counter-selection was done using synthetic complete medium containing uracil and 5-FOA at 1 mg·mL^−1^. Plasmids pSUP70-65-2 µ and pSUP70-65-CEN carry the *sup70-65* allele of *tQ(CUG)* and either the 2 µ origin or CEN-ARS, respectively [40].

### 3.2. Phenotypic Assays

Yeast strains were either grown on YPD or appropriate SC solid medium to select for the presence of plasmids for 24 to 36 h. Cells were recovered from the plates and resuspended in sterile water. Cell densities were measured using absorbance at 600 nm and dilutions prepared from this suspension with final OD_600nm_ values of 0.15, 0.015, 0.0015 and 0.00015. These were spotted on either drug-free solid YPD plates or YPD plates supplemented with sterile rapamycin at a final concentration of 5 nM. Subsequently, plates were incubated at different temperatures for 36–72 h and photographed. The longer incubation time (72 h) was used for phenotypic assay with *deg1 elp3* double mutants. For qualitative read-through of *ade2-1* and *trp1-1* alleles, serial dilution were spotted on SC lacking adenine or tryptophan respectively and incubated for 48 to 60 h at 30 °C.

### 3.3. Quantitative Read-through Assay

Cells carrying pUKC815 (wild type *lacZ*) or pUKC817 (UAA ochre insertion) [42] were grown at 30 °C or 37 °C in liquid SC medium lacking uracil to OD_600_ nm of 2–3 and harvested by centrifugation. Cells were washed and resuspended in Z-buffer (60 mM Na_2_HPO_4_, 40 mM NaH_2_PO_4_, 10 mM KCl, 50 mM 2-mercaptoethanol, pH 7). Cell density was measured by absorbance at 600 nm. To 500 µL aliquots, two drops of 0.01% sodium dodecyl sulfate (SDS) solution and chloroform were added and samples mixed on a vortex for 30 s each. Following incubation at 37 °C for 5 min, 100 µL of 4 mg·mL^−1^ ortho-nitrophenyl-β-galactoside dissolved in Z-buffer was added. Reactions were stopped by the addition of 250 µL 1 M Na_2_CO_3_ and absorbance at 420 nm measured. Activity units were calculated by employing Miller’s formula [48]. Relative read-through efficiency (%) was calculated by dividing the beta galactosidase activity measured with the pUKC817 construct by the one measured with the pUKC815 construct [33,42]. For each strain, at least three independent cultures were measured with both constructs.

## 4. Conclusions

The emerging picture from the presented work and previous studies places Deg1-mediated ψ38 in a role to maintain the function of both Gln isoacceptor tRNAs. However, the presence of ψ38 becomes critical for tRNA function only in the presence of an additional disturbance of the ASL structure. In one case (tRNA^Gln^_UUG_), a strong growth defect indicative of a significant loss of function is observed specifically when *deg1* mutants are shifted to conditions known to downregulate the formation of a distinct modification (mcm^5^s^2^U) (Figure 1 and Figure 2). In another case (tRNA^Gln^_CUG_) where this modification is absent per se, the introduction of a destabilization of the ASL can substitute for a hypomodified wobble base and sensitize this tRNA as well for the negative effects of the *deg1* mutation (Figure 3 and Figure 4). It appears possible that other tRNA species such as tRNA^Tyr^_GψA_ do not lose function in the absence of ψ38/39 due to the existence of additional modifications that may stabilize the ASL. For example, tRNA^Tyr^_GψA_-derived suppressor tRNAs are known to carry N^6^-isopentenyl-adenosine (i^6^A) in position 37 [7]. The presence of i^6^A has been shown to support efficient ochre read-through [49] and therefore may protect the ASL from destabilization. We assume that multiple modifications of the ASL provide multiple layers of structural protection, which routinely are not ruptured in single tRNA modification mutants and this functional redundancy may explain the paradox situation of missing phenotypes for many of the evolutionary conserved tRNA modification genes.

## Figures and Tables

**Table 1:** Strains used in this study.

Strain	Genotype	Reference/Source
*Saccharomyces cerevisiae* BY4741	MATa, *his3Δ, leu2Δ, met15Δ, ura3Δ*	Euroscarf, Frankfurt
*S. cerevisiae* elp3	BY4741 *elp3ΔKanMX4*	Euroscarf, Frankfurt
*S. cerevisiae* urm1	BY4741 *urm1ΔKanMX4*	Euroscarf, Frankfurt
*S. cerevisiae* deg1	BY4741 *deg1ΔKanMX4*	Euroscarf, Frankfurt
*S. cerevisiae* elp3 urm1	BY4741 *elp3ΔKanMX4 urm1ΔHIS3*	[31]
*S. cerevisiae* deg1 urm1	BY4741 *urm1ΔKanMX4 deg1ΔSpHIS5*	[9]
*S. cerevisiae* deg1 elp3	BY4741 *elp3ΔKanMX4 deg1ΔSpHIS5*	[9]
*S. cerevisiae* sup70	BY4741 pAK01 *tQ(CUG)ΔKlLEU2*	[31]
*S. cerevisiae* sup70-65-pAK01	BY4741 pAK01 *tQ(CUG)ΔKlLEU2* pSUP70-65-CEN	this work
*S. cerevisiae* sup70-65	BY4741 *tQ(CUG)ΔKlLEU2* pSUP70-65 CEN	this work
*S. cerevisiae* sup70-65-pAK01	BY4741 pAK01 *tQ(CUG)ΔKlLEU2 deg1ΔKanMX4* pSUP70-65-CEN	this work
*S. cerevisiae* W303-1B	MATα *leu2-3,112 trp1-1 can1-100 ura3-1 ade2-1 his3-11,15*	[47]
*S. cerevisiae* RK207	W303-1B *deg1ΔSpHIS5*	this work
*S. cerevisiae* RK273	W303-1B *deg1ΔloxP*	this work
*S. cerevisiae* UMY2893	MATα *SUP4* *leu2-3,112 trp1-1 can1-100 ura3-1 ade2-1 his3-11,15*	[10]
*S. cerevisiae* UMY2916	UMY2893 *elp3∆KanMX4*	[10]
*S. cerevisiae* RK208	UMY2893 *deg1ΔSpHIS5*	this work
